# Validation of an imageless electrocardiographic imaging technique for the non-invasive mapping of regular atrial tachyarrhythmias

**DOI:** 10.1093/europace/euag048

**Published:** 2026-03-12

**Authors:** Jana Reventos-Presmanes, Andrea Cano, Eric Invers-Rubio, Berta Pellicer-Sendra, Jaume Serrano-Campaner, Ernesto Zacur, Till F Althoff, Ismael Hernández-Romero, Clara Herrero-Martín, Roger Borràs, Mariona Regany-Closa, Elena Arbelo, Eduard Guasch, Jose María Tolosana, Andreu Porta-Sánchez, Ivo Roca-Luque, María S Guillem, Andreu M Climent, Lluís Mont, Jean-Baptiste Guichard

**Affiliations:** Institut Clínic Cardiovascular (ICCV), Hospital Clínic, Universitat de Barcelona, Cr. Villarroel 170, Catalonia 08036, Spain; ITACA Institute, Universitat Politècnica de València, Valencia, Spain; Corify Care SL, Madrid, Spain; ITACA Institute, Universitat Politècnica de València, Valencia, Spain; Corify Care SL, Madrid, Spain; Institut Clínic Cardiovascular (ICCV), Hospital Clínic, Universitat de Barcelona, Cr. Villarroel 170, Catalonia 08036, Spain; Institut Clínic Cardiovascular (ICCV), Hospital Clínic, Universitat de Barcelona, Cr. Villarroel 170, Catalonia 08036, Spain; Corify Care SL, Madrid, Spain; Institut Clínic Cardiovascular (ICCV), Hospital Clínic, Universitat de Barcelona, Cr. Villarroel 170, Catalonia 08036, Spain; Corify Care SL, Madrid, Spain; Corify Care SL, Madrid, Spain; Institut Clínic Cardiovascular (ICCV), Hospital Clínic, Universitat de Barcelona, Cr. Villarroel 170, Catalonia 08036, Spain; Department of Cardiology, Angiology and Intensive Care Medicine, German Heart Center of the Charité (DHZC), Berlin, Germany; ITACA Institute, Universitat Politècnica de València, Valencia, Spain; Corify Care SL, Madrid, Spain; ITACA Institute, Universitat Politècnica de València, Valencia, Spain; Institut Clínic Cardiovascular (ICCV), Hospital Clínic, Universitat de Barcelona, Cr. Villarroel 170, Catalonia 08036, Spain; Centro de Investigación Biomédica en Red e Salud Mental, CIBERSAM, Instituto de Salud Carlos III, Madrid, Spain; Institut Clínic Cardiovascular (ICCV), Hospital Clínic, Universitat de Barcelona, Cr. Villarroel 170, Catalonia 08036, Spain; Institut Clínic Cardiovascular (ICCV), Hospital Clínic, Universitat de Barcelona, Cr. Villarroel 170, Catalonia 08036, Spain; Institut D’Investigacions Biomèdiques August Pi I Sunyer (IDIBAPS), Barcelona, Spain; Centro de Investigación Biomédica en Red de Enfermedades Cardiovasculares (CIBERCV), Madrid, Spain; Institut Clínic Cardiovascular (ICCV), Hospital Clínic, Universitat de Barcelona, Cr. Villarroel 170, Catalonia 08036, Spain; Institut D’Investigacions Biomèdiques August Pi I Sunyer (IDIBAPS), Barcelona, Spain; Centro de Investigación Biomédica en Red de Enfermedades Cardiovasculares (CIBERCV), Madrid, Spain; Institut Clínic Cardiovascular (ICCV), Hospital Clínic, Universitat de Barcelona, Cr. Villarroel 170, Catalonia 08036, Spain; Institut D’Investigacions Biomèdiques August Pi I Sunyer (IDIBAPS), Barcelona, Spain; Centro de Investigación Biomédica en Red de Enfermedades Cardiovasculares (CIBERCV), Madrid, Spain; Institut Clínic Cardiovascular (ICCV), Hospital Clínic, Universitat de Barcelona, Cr. Villarroel 170, Catalonia 08036, Spain; Institut D’Investigacions Biomèdiques August Pi I Sunyer (IDIBAPS), Barcelona, Spain; Institut Clínic Cardiovascular (ICCV), Hospital Clínic, Universitat de Barcelona, Cr. Villarroel 170, Catalonia 08036, Spain; Institut D’Investigacions Biomèdiques August Pi I Sunyer (IDIBAPS), Barcelona, Spain; Centro de Investigación Biomédica en Red de Enfermedades Cardiovasculares (CIBERCV), Madrid, Spain; ITACA Institute, Universitat Politècnica de València, Valencia, Spain; Corify Care SL, Madrid, Spain; ITACA Institute, Universitat Politècnica de València, Valencia, Spain; Corify Care SL, Madrid, Spain; Institut Clínic Cardiovascular (ICCV), Hospital Clínic, Universitat de Barcelona, Cr. Villarroel 170, Catalonia 08036, Spain; Institut D’Investigacions Biomèdiques August Pi I Sunyer (IDIBAPS), Barcelona, Spain; Centro de Investigación Biomédica en Red de Enfermedades Cardiovasculares (CIBERCV), Madrid, Spain; Institut Clínic Cardiovascular (ICCV), Hospital Clínic, Universitat de Barcelona, Cr. Villarroel 170, Catalonia 08036, Spain; Institut D’Investigacions Biomèdiques August Pi I Sunyer (IDIBAPS), Barcelona, Spain; National Heart and Lung Institute, Hammersmith Campus, Imperial College London, London, UK

**Keywords:** AFL: atrial flutter, AT: atrial tachycardia, EAM: electroanatomical mapping, ECGi: electrocardiographic imaging, CT: computed tomography, MRI: magnetic resonance imaging

## Introduction

Regular atrial tachyarrhythmias, including atypical atrial flutter (AFL) and focal atrial tachycardia (AT), are common,^1^ particularly following ablation of atrial fibrillation (AF). While typical AFL can often be identified on a 12-lead electrocardiogram (ECG), determining the mechanism and location of other atrial arrhythmias remains challenging.^2^ Electroanatomical mapping (EAM) is the gold standard but is invasive and requires sustained arrhythmia.^3^ Classical electrocardiographic imaging (ECGi) offers a promising non-invasive alternative,^4^ though its reliance on pre-acquired cardiac imaging^5^ [computed tomography (CT) or magnetic resonance imaging (MRI)], lengthy setup, and limited inverse reconstruction restricts its use.^6,7^ Recently, an imageless ECGi system enabling non-invasive mapping without prior imaging has been introduced,^8,9^ but its clinical performance in atrial tachyarrhythmias has not been systematically evaluated. This study assessed the anatomical accuracy and diagnostic value of imageless ECGi compared with classical ECGi and EAM.

## Methods

### Study population

A total of 68 patients with focal AT or AFL were prospectively enrolled (mean age 61 years; 63% male; 49% prior ablation; mean left atrium area 30 cm^2^). Patients were assigned either to a comparison cohort (*n* = 25) with available CT/MRI for comparison of classical and imageless ECGi or to a validation cohort (*n* = 43) for independent assessment. The study was approved by the local ethics committee, and all patients provided informed consent.

### Electrocardiographic imaging acquisition

All patients underwent simultaneous 12-lead ECG, non-invasive ECGi (ACORYS, Corify Care SL), and EAM during the clinical procedure. Following electrode placement, a 3D torso scan was acquired.^9^ When atrial activity was not identifiable, vagal manoeuvres were performed to increase the R-R interval. In the imageless ECGi workflow, the torso scan was fitted to a statistical shape model (SSM) trained on a large CT/MRI database capturing torso–atria variability.^8,10^ The algorithm iteratively aligned the scan to the most probable SSM torso-atria geometry, minimizing surface mismatch. In classical ECGi, patient-specific CT/MRI images were segmented (ADAS3D Medical SL) and co-registered to the torso scan using an iterative closest-point algorithm. In both methods, body-surface potentials were inverted to reconstruct atrial electrograms (EGMs) and compute local activation time (LAT) maps as previously described.^10^

### Evaluation metrics

Anatomical accuracy of imageless ECGi was quantified in the comparison cohort establishing vertex correspondence between meshes with consistent surface points and anatomical atrial landmarks to assess mean atrial surface distance between estimated and true geometries. Electrophysiological differences between classical and imageless ECGi maps were computed using correlation coefficient (CC) for EGMs morphology and mean absolute error (MAE), expressed in milliseconds, for LAT values. Clinical accuracy was assessed against invasive EAM for three predefined endpoints: (i) arrhythmia chamber, (ii) mechanism (focal or macro-re-entry), and (iii) ablation target (earliest activation or critical isthmus). Analyses were performed blinded to both surface ECG and EAM.

## Results

### Atrial arrhythmia characterization

A total of 79 arrhythmias were analysed: 30 (38.0%) in the comparison cohort and 49 (62.0%) in the validation cohort. The diagnosis based on EAM included 44 atypical AFL, 23 typical AFL, and 12 focal AT. Three cases were excluded from the analysis due to poor signal quality or unsuccessful manoeuvres failure that prevented acquisition of a complete atrial activation window.

### Comparison of imageless vs. classical electrocardiographic imaging

**Figure 1:**
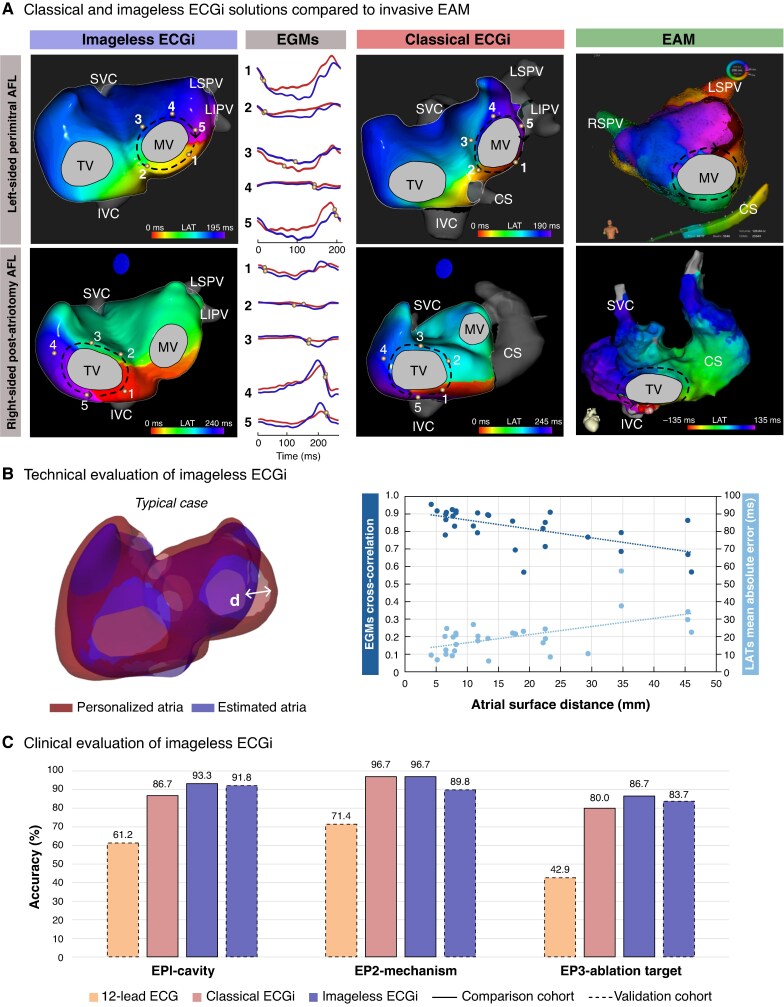
Imageless ECGi for the non-invasive mapping of regular atrial tachyarrhythmias. (*A*) Clinical cases of a left-sided (top) and right-sided (bottom) atrial flutters comparing imageless and classical ECGi approaches with the gold-standard EAM. Representative inverse atrial EGMs around the re-entrant circuits are displayed for both methods. (*B*) Technical evaluation of imageless ECGi. *Left side:* representative example of typical scenarios showing personalized and estimated atria meshes. *Right side:* relationship between atrial surface distance (x-axis), EGM morphology cross-correlation (left y-axis), and LAT mean absolute error in milliseconds (right y-axis) compared to classical ECGi solutions for all maps evaluated in the comparison cohort. (*C*) Diagnostic capacities of imageless ECGi compared to classical ECGi and traditional 12-lead ECG regarding the characterization of complex atrial arrhythmia for the comparison (continuous line) and validation (dashed line) cohorts. AFL, atrial flutter ; CS, coronary sinus; EAM, electroanatomical mapping; ECGi, electrocardiographic imaging; EGMs, electrograms; EP, endpoint, IVC, inferior vena cava; LATs, local activation times; LIPV, left inferior pulmonary vein; LSPV, left superior pulmonary vein; MV, mitral valve; RSPV, right superior pulmonary vein; SVC, superior vena cava; TV, tricuspid valve.

*Figure 1A* shows representative examples from the comparison cohort of complex AFL using the imageless and classical ECGi approaches compared to EAM. The technical performance of imageless ECGi is shown in *Figure 1B*, which illustrates a representative atrial geometry estimation compared with the MRI-based geometry. Additionally, the panel shows the relationship between geometric accuracy, the CC of EGM morphology, and the MAE of LATs. Overall, the mean surface distance between the estimated and personalized geometries was 15.3 ± 10.2 mm. The average CC for EGM morphology was 0.8 ± 0.1, and the average MAE for LATs was 20.0 ± 10.5 ms. The variability in surface distance had a moderate impact on these electrophysiological parameters. For the comparison cohort, both imageless and classical ECGi demonstrated comparable diagnostic accuracy as shown in *Figure 1C*; this included the identification of the cavity involved (93.3% vs. 86.7%, *P* = 0.7), the arrhythmia mechanism (96.7% vs. 96.7%, *P* = 1.0), and the ablation target (86.7% vs. 80.0%, *P* = 0.2).

### Predictive capacity of imageless electrocardiographic imaging

In the validation cohort, imageless ECGi maintained a clinical diagnostic accuracy comparable to EAM and significantly outperformed 12-lead ECG in identifying the involved cavity (91.8% vs. 61.2%, *P* < 0.001), arrhythmia mechanism (89.8% vs. 71.4%, *P* < 0.05), and ablation target (83.7% vs. 42.9%, *P* < 0.001), as shown in *Figure 1C*. Specifically, in the detection of re-entry mechanisms, Imageless ECGi demonstrated higher diagnostic performance compared to 12-lead ECG, with 94.6% vs. 73.7% sensitivity and 100% vs. 87.5% specificity, respectively. The diagnostic accuracy of imageless ECGi remained consistent for non-counterclockwise typical AFL (*n* = 38) and in patients with prior ablation (*n* = 27), with 12-lead ECG significantly less accurate for chamber and target localization. Additionally, accurate pre-procedural identification of the right atrium as the involved cavity could have avoided six transseptal punctures, accounting for 24% of the 25 transseptal approaches.

## Discussion

This study demonstrates that accurate non-invasive ECGi of regular atrial tachyarrhythmias can be achieved without cardiac imaging. Despite introducing measurable geometric differences, imageless ECGi maintained diagnostic accuracy and clinical interpretability comparable to classical ECGi.

### Diagnostic equivalence despite anatomical differences

The mean atrial surface distance (∼15 mm) between imageless and CT-/MRI-derived geometries lies within the physiological range of atrial displacement during the cardiac cycle. Consequently, imageless ECGi preserves sufficient spatial fidelity to generate meaningful inverse solutions and LAT maps, enabling reliable arrhythmia characterization. These findings indicate that absolute anatomical precision relative to a static pre-procedural image is not required for accurate functional mapping. High CC for EGMs and moderate MAE for LATs were maintained even in patients with surface distances exceeding 30 mm. These discrepancies likely reflect physiological phenomena, such as respiratory motion and cardiac contraction, which are inherently present both when performing rigid registration to a static CT/MRI scan and in ‘imageless’ torso-to-heart approaches. The diagnostic disagreement observed is more plausibly explained by physiological motion or complex substrate characteristics rather than geometric factors.

### Clinical and practical advantages

By eliminating the need for CT/MRI, imageless ECGi reduces preparation time, cost, and radiation exposure while minimizing dependence on imaging infrastructure. The acquisition requires only a torso scan that can be completed within minutes, allowing both pre-procedural and intra-procedural non-invasive mapping. Compared with the standard ECG, imageless ECGi provided superior diagnostic precision, particularly for chamber and ablation-target prediction. Its ability to identify right atrial tachyarrhythmias prior to mapping suggests potential to streamline procedural strategy and avoid unnecessary transseptal puncture. Furthermore, the imaging-free setup enables single-beat mapping of transient or non-sustained arrhythmias, a frequent scenario during ablation of persistent AF where conversion to AFL often occurs before sinus rhythm.

## Conclusion

This study validates the clinical performance of an imageless ECGi system for the characterization of regular atrial tachyarrhythmias. Its ability to provide rapid, full-chamber activation maps from a single beat may make it a potential solution for mapping transient arrhythmias or rhythm changes during ablation procedures.

## Data Availability

The data underlying this article will be shared on reasonable request to the corresponding author.
